# Hybrid surgical and endovascular management of severe gastrointestinal hemorrhage from mesenteric arteriovenous malformation

**DOI:** 10.1016/j.jvscit.2025.102083

**Published:** 2025-12-08

**Authors:** Tuong-Anh Mai-Phan, Hien Minh Tran, Ngoc Son Vu, Duc Chi Tieu, Khanh-Phat Thai, Kim-Long Le

**Affiliations:** a Department of Hepato-Pancreato-Biliary Surgery, Nhan dan Gia Dinh Hospital, Ho Chi Minh City, Vietnam; b Department of Radiology, Nhan dan Gia Dinh Hospital, Ho Chi Minh City, Vietnam; c Department of Gastrointestinal Surgery, Nhan dan Gia Dinh Hospital, Ho Chi Minh City, Vietnam; d Department of Thoracic and Vascular Surgery, Nhan dan Gia Dinh Hospital, Ho Chi Minh City, Vietnam; e Department of Surgery, Pham Ngoc Thach University of Medicine, Ho Chi Minh City, Vietnam

**Keywords:** Arteriovenous malformation, Embolization, Gastrointestinal hemorrhage, Portal hypertension, Portosystemic shunt

## Abstract

Mesenteric arteriovenous malformation is a rare vascular anomaly that can worsen portal hypertension and cause life-threatening gastrointestinal bleeding. We report a 37-year-old man with chronic portal and mesenteric vein thrombosis who presented with recurrent hematochezia refractory to endoscopic and medical therapy. Imaging demonstrated diffuse mesenteric arteriovenous malformation with venous congestion. A staged hybrid strategy was performed: selective embolization reduced shunt flow, followed by bowel resection and creation of a mesocaval shunt. Histopathology confirmed extensive venous abnormalities. The patient recovered uneventfully, and no recurrent bleeding occurred during 9 months of follow-up.

Mesenteric arteriovenous malformation (AVM) is an uncommon high-flow vascular lesion of the splanchnic circulation that produces arteriovenous shunting, which in turn drives venous hypertension, mucosal ischemia through a steal phenomenon, and gastrointestinal bleeding.^1^^,^^2^ Diagnosis relies on cross-sectional angiographic imaging to define the angio-architecture and guide therapy, most often contrast-enhanced computed tomography and mesenteric angiography.^2^^,^^3^ Endovascular embolization can be effective, particularly when treatment is tailored to the angio-architecture, although diffuse or recurrent disease may require surgical resection.^2^^,^^4^^,^^5^ Splanchnic AVMs and arteriovenous fistulas can exacerbate portal hypertension and, in select cases, demand combined or staged approaches.^6^ When chronic portal vein thrombosis with cavernous transformation coexists, management must also address portal decompression; surgical portosystemic shunts such as a mesocaval shunt are established options when a transjugular intrahepatic portosystemic shunt is not feasible.^7^ We report a young adult with life-threatening lower gastrointestinal bleeding due to diffuse mesenteric AVM in the setting of chronic portal vein thrombosis with cavernous transformation, successfully treated with a deliberately staged hybrid strategy comprising targeted embolization, bowel resection, and a mesocaval portosystemic shunt, resulting in durable bleeding control.

## Case report

A 37-year-old man with a history of chronic portal and superior mesenteric vein thromboses for more than 10 years presented with recurrent hematochezia, presyncope, and fatigue. At admission, he was hemodynamically unstable, with hemoglobin of 6.2 g/dL, mild hyponatremia, and transient kidney injury. Despite fluid resuscitation and prior endoscopic therapy, bleeding persisted.

**Fig 1:**
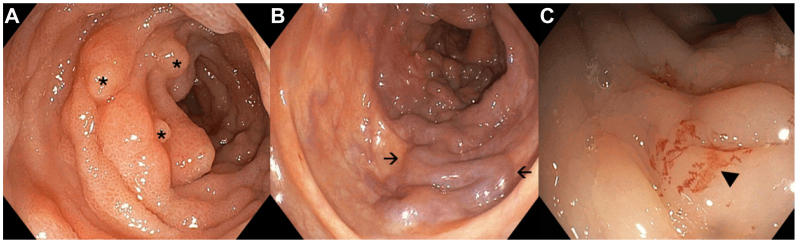
Colonoscopy findings: Initial colonoscopy revealed widespread mucosal abnormalities without active bleeding. **(A)** Multiple scattered polyps throughout the colonic lumen (indicated by *asterisks*). **(B)** Dilated, tortuous veins protruding into the colonic lumen in the descending colon, with mucosal edema and pseudomembrane formation (indicated by *arrows*). Hemostatic clips from prior interventions are also noted. **(C)** Distal ileum with edematous mucosa and subtle mucosal bleeding without progression (indicated by *arrowhead*).

**Fig 2:**
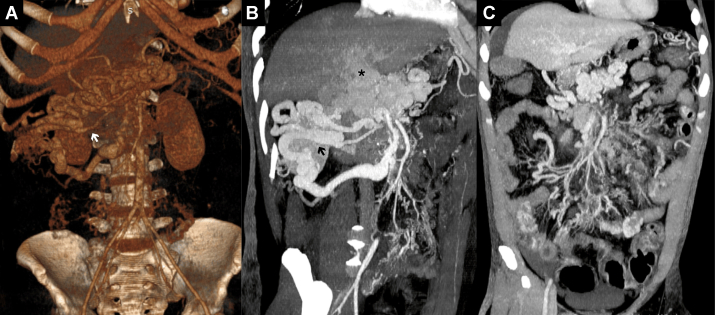
Computed tomography findings of chronic portal vein thrombosis with cavernous transformation and mesenteric arteriovenous malformation (AVM). **(A)** Three-dimensional reconstruction shows cavernous transformation at the hepatic hilum with dilated mesenteric veins (*arrow*). **(B)** Arterial-phase sagittal computed tomography demonstrates dilated mesenteric branches (*arrow*) and diffuse AVM at the hilum (*asterisk*) and mesentery. **(C)** Coronal venous-phase computed tomography reveals tortuous, dilated mesenteric veins consistent with venous congestion and arteriovenous fistulas.

**Fig 3:**
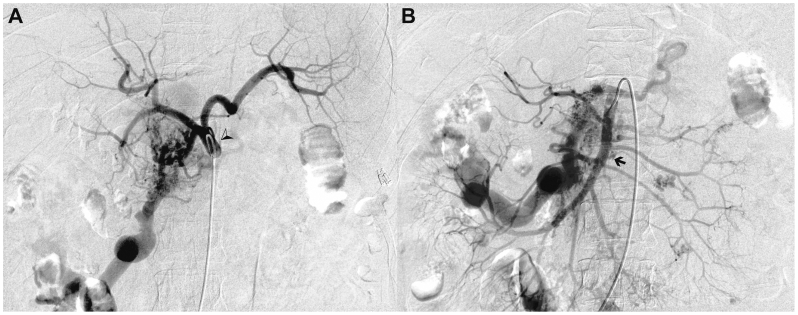
Mesenteric arteriovenous malformation (AVM). The mesenteric AVM consisted of numerous abnormally developed small arteries originating from the celiac trunk and superior mesenteric artery systems. **(A)** Digital subtraction angiography with selective catheterization of the celiac trunk (*arrowhead*), showing multiple abnormal small arteries originating from the celiac trunk with abnormal vascular remodeling consistent with AVM. **(B)** Digital subtraction angiography with selective catheterization of the superior mesenteric artery (*arrow*), demonstrating abnormal vascular networks and AVM involving branches of the superior mesenteric artery.

Colonoscopy showed congestive colopathy with venous ectasia, edematous mucosa, superficial ulcerations, and diffuse oozing without a discrete endoscopic target (Fig 1, *A-C*). Contrast-enhanced computed tomography demonstrated chronic portal and superior mesenteric vein thrombosis with cavernous transformation and markedly dilated mesenteric venous branches, compatible with a diffuse high-flow mesenteric AVM (Fig 2, *A-C*). Selective angiography identified a large AVM with multiple hypertrophic feeding arteries arising from the right gastric artery, the gastroduodenal artery, and branches of the superior mesenteric artery. Venous outflow occurred predominantly through a dilated ileocolic and inferior mesenteric vein with flow reversal into peri intestinal and submucosal venous plexuses and extensive collateralization to the portal venous system, resulting in secondary portal hypertension (Fig 3, *A* and *B*). Although quantitative flow measurements were not obtained, digital subtraction angiography demonstrated immediate arteriovenous shunting with rapid venous opacification. Transthoracic echocardiography showed no evidence of high-output cardiac failure, and cardiopulmonary status remained stable.

**Fig 4:**
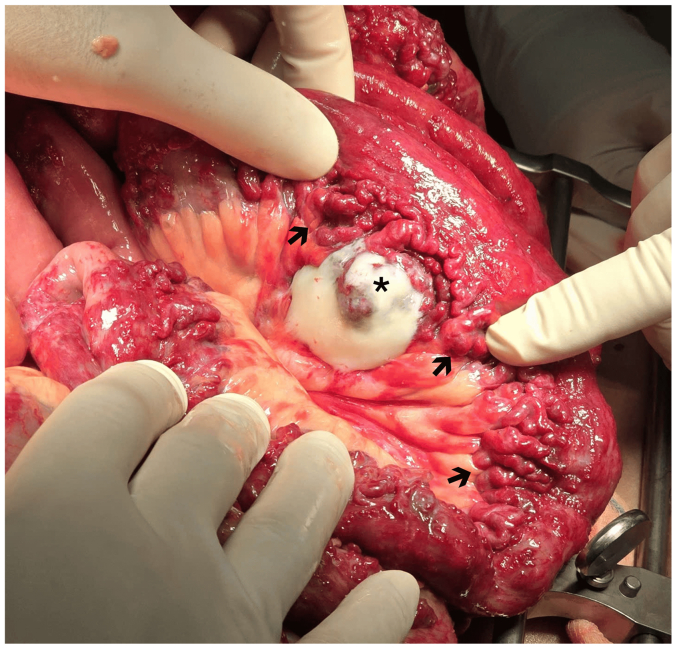
Exploratory laparotomy revealed tortuous, dilated mesenteric veins. A 3-meter small intestinal segment showed severe venous congestion due to arteriovenous malformations (AVMs) (*arrows*), and an ulcerative lesion (*asterisk*) was histopathologically confirmed as an angiolipoma adjacent to the intestinal wall.

Given the refractory bleeding and diffuse shunt burden, a staged hybrid approach was adopted. Step 1 (digital subtraction angiography suite), via right common femoral arterial access (Seldinger technique), the celiac trunk and superior mesenteric artery were selectively catheterized. Targeted transarterial embolization of the celiac feeders (right gastric and gastroduodenal branches) was performed using a coil-assisted N-butyl 2-cyanoacrylate technique, achieving stable occlusion while preserving mesenteric perfusion. Embolization of superior mesenteric branches was deferred to avoid bowel ischemia. Step 2 (operating room under mobile C arm), at laparotomy, markedly dilated mesenteric veins and a ∼3 meter segment of congested small bowel with mucosal ulcerations were identified and resected with a functional end-to-end anastomosis (Fig 4). The ileocolic vein was accessed for intraoperative venography, and an Amplatzer Vascular Plug II with adjunctive coils achieved complete occlusion of the dominant venous outflow. To decompress the hypertensive mesenteric venous system, a side-to-side mesocaval portosystemic shunt was created between the inferior mesenteric vein and the inferior vena cava with an anastomotic diameter of approximately 1 cm, resulting in immediate improvement in bowel perfusion. Although the two steps were performed in separate settings, the interventions were designed as a staged hybrid strategy, integrating endovascular flow reduction and open surgical decompression within a single coordinated therapeutic plan.

Histopathology showed markedly dilated submucosal, muscular, and serosal veins with prominent congestion, and resection margins free of residual malformation. A benign angiolipoma adjacent to the intestinal wall was identified. Germline testing revealed a heterozygous missense variant in the PTEN gene (NM_000,314.8: c.464 A > G; NP_000,305.3: p.Tyr155Cys, located at chr10:87,933,223), classified as pathogenic and consistent with PTEN hamartoma tumor syndrome.

The postoperative course was uneventful. Doppler ultrasonography confirmed shunt patency, and nutritional rehabilitation was initiated early. At 10-month follow-up, the patient remained free of recurrent gastrointestinal bleeding.

Written informed consent for publication of the case details and associated images was obtained from the patient, with all identifying information removed.

## Discussion

This case highlights the hemodynamic complexity of mesenteric AVM on chronic portal vein thrombosis with cavernous transformation. High-flow shunting raises mesenteric venous pressure and diverts arterial inflow, producing venous hypertension and ischemia that predispose to bleeding. Mesenteric arteriovenous communications can worsen portal hypertension and present with severe hemorrhage or high-output states, emphasizing the need for definitive flow reduction rather than supportive care.^6^

A staged hybrid strategy offered sequential control. Targeted embolization reduced shunt flow, limited intraoperative bleeding, and clarified residual disease. Reports indicate embolization tailored to the angioarchitecture can lower portal pressure and improve outcomes in mesenteric AVM. Reference to the Yakes classification assists in selecting access and embolic targets.^8^^,^^9^ In our patient, persistent congestion and ulcerated bowel required resection to remove diseased intestine and bleeding sites unlikely to heal under hypertension.

Portal decompression was a second cornerstone. In chronic portal vein thrombosis with cavernoma, transjugular intrahepatic portosystemic shunt is often difficult and relatively contraindicated, although recanalization techniques are emerging. When transhepatic or transjugular options are not feasible, a mesocaval shunt provides prehepatic decompression and protects the anastomosis from venous hypertension.^10^^,^^11^ The absence of bleeding at 9 months supports the durability of combining source control with venous pressure reduction.

The genetic finding adds mechanistic context. The patient carried a heterozygous PTEN missense variant NM_000,314.8:c.464 A > G (p.Tyr155Cys), which is classified as pathogenic for PTEN hamartoma tumor syndrome by expert curation and ClinVar submissions. PTEN hamartoma tumor syndrome encompasses a spectrum of mucocutaneous, neoplastic, and vascular manifestations; vascular anomalies, including arteriovenous malformations and venous malformations, are well-documented features.^12^^,^^13^ Recent series also highlight AVMs as a presenting sign of the syndrome, underscoring the value of genetic evaluation when complex or multifocal vascular malformations are encountered.^14^ Mechanistic work supports a causal role for endothelial PTEN loss in driving PTEN hamartoma tumor syndrom-related vascular malformations, which aligns with the phenotype seen here.^15^ Notably, the same p.Tyr155Cys variant has been independently reported as pathogenic in the clinical genetics literature, strengthening the inference that constitutional PTEN dysfunction contributed to the vascular phenotype in this patient.^16^

Taken together, three practical lessons emerge. First, diffuse mesenteric AVM in a background of fixed portal hypertension often requires combined endovascular and surgical therapy, sequenced to balance ischemia risk against undertreatment. Second, when transjugular intrahepatic shunting is not feasible because of chronic portal vein thrombosis with cavernomatous transformation, mesocaval decompression is a reasonable alternative. Third, identifying a pathogenic PTEN variant provides an explanatory framework for the vascular disease and triggers syndrome-appropriate surveillance for associated neoplasia and additional vascular anomalies.^12^

## Conclusions

This case represents the first reported instance in Vietnam of severe gastrointestinal hemorrhage caused by idiopathic portal hypertension associated with mesenteric AVM, successfully managed through a hybrid surgical and endovascular approach. The rarity of this condition, compounded by its complex pathophysiology and diagnostic challenges, underscores its clinical significance. Although similar cases have been documented globally, this report highlights a novel combination of interventional radiology-guided embolization and surgical resection, tailored to the intricate vascular anatomy and hemodynamic compromise.

## Funding

None.

## Disclosures

None.
